# Prevalence, associated factors and clinical implications of medication literacy linked to frailty in hemodialysis patients in China: a cross-sectional study

**DOI:** 10.1186/s12882-023-03346-4

**Published:** 2023-10-24

**Authors:** Linfang Zhu, Yang Liu, Fengxue Yang, Shaobin Yu, Ping Fu, Huaihong Yuan

**Affiliations:** 1 Department of Nephrology, Kidney Research Institute, West China Hospital of Sichuan University, Chengdu, 610041 China; 2 West China School of Nursing, Sichuan University, Chengdu, 610041 China; 3 Sichuan Nursing Vocational College, Chengdu, 610041 China

**Keywords:** Hemodialysis, Medication literacy, Frailty

## Abstract

**Background:**

Maintenance hemodialysis (MHD) patients have complex medication regimens that require a high level of skill to interpret medication information. However, there is currently a lack of research evaluating the ability to read and understand medication labels in Chinese MHD patients. In addition, the relationship between frailty and medication literacy among MHD patients remains unclear. Therefore, this study aims to assess the potential factors affecting medication literacy in MHD patients and to explore the relationship between frailty and medication literacy.

**Methods:**

This cross-sectional study was conducted using convenience sampling in West China Hospital of Sichuan University, China. Using a general questionnaire, we collected demographic, clinical and laboratory data. Medication literacy was assessed by the Chinese Medication Literacy Scale, and frailty was assessed by the FRAIL Scale. Univariate analyses examined potential factors associated with medication literacy. An ordered logistic regression was used to analyze the relationships between medication literacy and these factors. Spearman’s correlation was used to assess the association between medication literacy and frailty.

**Results:**

A total of 290 MHD patients were included in the analysis. Inadequate, marginal, and adequate medication literacy was found in 56 (19.3%), 153 (52.8%), and 81 (27.9%) patients, respectively. Ordered logistic regression revealed factors associated with inadequate medication literacy: age (OR = 0.281, 95% CI = 0.139–0.565, p < 0.001 for < 65 years); education (OR = 8.612, 95% CI = 3.524–21.046, p < 0.001 for ≤ primary school education; OR = 3.405, 95% CI = 1.683–6.887, p = 0.001 for junior high school education); presence of caregiver medication assistance (OR = 2.302, 95% CI = 1.173–4.516, p = 0.015); frailty (OR = 0.440, 95% CI = 0.216–0.893, p = 0.023 for frail patients); and high β2-microglobulin (β2-MG) (OR = 1.010, 95% CI = 1.002–1.019, p = 0.012). Spearman’s analysis showed that medication literacy was negatively correlated with frailty in MHD patients (R=-0.189, p = 0.001).

**Conclusions:**

Medication literacy levels in MHD patients needed improvement and were associated with certain patient characteristics, including age, education level, presence of caregiver support, β2-microglobulin levels, and risk of frailty. This study identified subgroups of MHD patients, such as those who were older, had lower education, had caregiver assistance, had high β2-microglobulin levels, or were frail, to have inadequate medication literacy. These findings underscore the need for routine screening and targeted interventions to improve medication literacy in this population.

## Introduction

Patients with end-stage renal disease (ESRD) who receive hemodialysis often experience many comorbid conditions, such as anemia, hypertension, secondary hyperthyroidism, and electrolyte disturbances [1, 2]. These comorbidities were complex and required long-term oral administration of multiple drugs (consuming ≥ 5 medications or 12 doses per day) [3]. Polypharmacy is common among patients receiving hemodialysis [4]. However, medication nonadherence was also very common among ESRD patients receiving hemodialysis, with an average prevalence of 52.5% [5].

Since maintenance hemodialysis (MHD) patients had multiple comorbid illnesses and dialysis-associated complications, at least five or more medications were needed. Poor adherence meant that those patients needed to have the ability to read medication labels and instructions to control and prevent disease progression.

Medication literacy refers to an individual’s ability to obtain, understand, evaluate medication information and apply this information to make correct medication decisions and behaviors [6], which is an important factor for predicting accurate medication behavior. MHD patients have complex medication regimens that require substantial skills to interpret medication information and take medications accurately as prescribed.

A U.S. study found that MHD patients aged 65 and over had difficulty reading medication labels. Although most MHD patients were taking phosphate binders, only 16% of participants were able to correctly answer questions about the indications of phosphate binders [7]. The implications of this study were that routine assessment and screening of medication literacy abilities should be implemented for hemodialysis patients, especially the elderly. Targeted interventions were needed to improve their medication label reading skills and comprehension of medication uses. The findings underscored the urgency and importance of improving medication literacy among hemodialysis patients to ensure appropriate medication use and safety. Overall, this study showed that good medication literacy was an important guarantee for safe medication use and was vital to the health of MHD patients [7].

Accumulating studies have shown that age, gender, education, and economic status can affect people’s ability to understand and use medication information [8–10]. Elderly patients with multiple health conditions and frequent medication use are high-risk groups with low medication literacy [11, 12]. It has been reported that family guidance can help improve medication management [13].

Previous studies have shown that patients at risk of malnutrition have lower medication literacy levels than those with normal nutritional status [14]. A study in China found that medication literacy was a factor affecting frailty in elderly patients with heart disease. People with low medication literacy were 2.759 times more likely to be frail than those with adequate medication literacy (OR = 2.759, P < 0.001) [15]. This may be due to the poor compliance and adherence caused by low medication literacy, which may increase the risk of malnutrition and cause frailty. However, the relationship between medication literacy and frailty in MHD patients has not been reported before.

As modern clinical medical systems record much health and medical data and MHD patients undergo regular dialysis and centralized examinations, we wanted to explore whether there was an association between patients’ laboratory examinations and medication literacy.

Therefore, the aim of this study was to assess medication literacy and its influencing factors in MHD patients. We further explored the relationship between frailty and medication literacy.

## Methods

### Design

A descriptive cross-sectional study with analytical components was conducted.

#### Setting and participants

This cross-sectional study was conducted using convenience sampling. Data were collected from March 28, 2023, to May 31, 2023. Inclusion criteria were (1) clear consciousness, normal cognition, and no communication barriers; (2) receiving MHD treatment for at least 3 months; and (3) voluntary participation in the survey. The exclusion criteria were as follows: (1) unconsciousness, dementia, or mental illness; (2) communication barriers; and (3) current or previous engagement in medical and health-related work before retirement. The sample size was calculated using the formula N = Zα2P(1-P)/d2, where α represents the tolerance error, set at 0.05, and Zα = 1.96. The allowable error (d) was set at 3%.

A literature search [13] revealed that the prevalence of inadequate medication literacy among MHD patients in China was estimated to be 6.8%, resulting in a calculated sample size of 271. To account for invalid questionnaires, which were expected to constitute 5% of the total cases, the required sample size was adjusted to 285 cases.

### Data collection and procedures

Data collection was conducted during the dialysis process at the Wenjiang Hemodialysis Center in the Department of Nephrology at West China Hospital, Sichuan University, Chengdu, China. Two researchers (Yang Liu and Linfang Zhu) completed the data collection through one-on-one face-to-face interviews. After signing the informed consent form, participants completed the questionnaire. In illiterate patients, the consent was read to them in the presence of a literate relative and they provided a fingerprint on the consent form to indicate their informed consent to participate. If a participant could not fill out the questionnaire because their dominant arm was being used for dialysis, the interviewers read out the questionnaire content and provided assistance. Specifically, the questionnaire was administered mainly through interviewer assessment. For patients who were capable of self-administering, they completed the questionnaire by themselves. For those who had difficulties in writing, the interviewers helped them complete the questionnaire. We adopted this approach to ensure the quality of the data collected. Once the survey was completed, researchers immediately verified and collected the questionnaires. At the end of the survey, laboratory test results were obtained through queries in the laboratory test system from the patient’s most recent centralized examination at the hemodialysis center.

### Measures

The demographic characteristics collected in this study included gender, age, height, body weight, education level, monthly income, primary illness, whether medication was taken with the help of a caregiver, dialysis vintage, number of medications, and comorbidity. Evidence showed that BMI and biochemical indicators such as albumin, electrolytes, and total iron binding capacity were part of nutritional assessment [16]. β2-microglobulin reflected the level of middle molecule toxins in the MHD patient’s body. Effective removal of middle and large molecule toxins could improve the patient’s microinflammatory state and improve nutritional status [17]. Thus, the data collection also included laboratory findings obtained through the hospital Laboratory Information System from the latest centralized examination at the hemodialysis center. These findings included routine blood examinations such as red blood cell count (RBC), hemoglobin (HGB), albumin (ALB), ferritin (FER), β2-MG, predialysis creatinine (pre-CRE), serum calcium (s-Ca), serum inorganic phosphorus (s-IP), serum potassium (s-K), and parathyroid hormone (PTH). The main results of this study were obtained using two Chinese questionnaires: the Chinese Version of the Medication Literacy Scale and the FRAIL scale.

The level of medication literacy was assessed using the Chinese version of the Medication Literacy Scale, which included 14 items and consisted of four simulated drug use scenarios [18]. The specific questionnaire items could be referred to in previous studies by Qu et al. [14, 15]. Scenario 1 involved injectable medication for diabetics, Scenario 2 was about medication for children, Scenario 3 was on cold medicine, and Scenario 4 covered OTC and prescription drug. The total score was 14, with each of the 14 items representing one point. A score of 1 was given for a correct response, and a score of 0 was given for an incorrect response. The scores for each item were added to calculate the total score of the questionnaire. There were three levels of medication literacy: “adequate drug literacy”, “marginal medication literacy”, “inadequate medication literacy”. A total score of 11 or higher indicated adequate drug literacy, a total score between 4 and 10 indicated marginal medication literacy, and a total score of 3 or lower indicated inadequate medication literacy. The Chinese version of the Medication Literacy Scale demonstrated good reliability and validity, with a retest reliability of 0.885 and a split-half reliability of 0.840. The correlations between medication literacy and the corresponding items ranged from 0.427 to 0.587. The scale could be used to assess medication literacy among Chinese people. The Cronbach’s α coefficient measured in this study was 0.854.

The Frail Scale was utilized to evaluate frailty [19]. This scale included five self-reported questions (see Table 1). Each question was scored as 1 for a “yes” response and 0 for a “no” response, with total scores ranging from 0 to 5. A score of 0 indicated nonfrailty, a score of 1 to 2 indicated prefrailty, and a score of 3 or higher indicated frailty. The scale could be used to screen for frailty in patients on hemodialysis and proved to be an easy-to-apply tool [20].

**Table 1:** Demographic characteristics of HD (N = 290)

Characteristics	Groups	N (%)
Sex	Male	169 (58.28%)
	Female	121 (41.72%)
Age	<65	234 (80.69%)
	≥ 65	56 (19.31%)
Education level	Primary school or lower	59 (20.34%)
	Junior high school	94 (32.41%)
	Senior high school	62 (21.38%)
	University or higher	75 (25.86%)
Monthly income	<3000	109 (37.59%)
	3000 ~ 8000	129 (44.48%)
	>8000	52 (17.93%)
Primary illness	Hypertension	118 (40.69%)
	Diabetes	44 (15.17%)
	Others	96 (33.10%)
	Unknown	32 (11.03%)
Take medication with the help of a caregiver	Yes	54 (18.62%)
	No	236 (81.38%)
Dialysis vintage (yrs)	<1	36 (12.41%)
	1–5	124 (42.76%)
	>5	130 (44.83%)
No. of medications	< 5	171 (58.97%)
	≥ 5	119 (41.03%)
Comorbidity	< 5	213 (73.45%)
	≥ 5	77 (26.55%)

### Ethical considerations

This study was approved by the biomedical ethics committee of the West China University of Sichuan University (IRB NO.2023 − 362).

### Statistical analysis

SPSS version 25.0 was used for statistical analysis. Descriptive statistics were used to describe the patients’ baseline characteristics, frailty, and medication literacy. When the distribution of responses was approximately balanced across categorical variable levels, the chi-squared test or Fisher’s exact probability method was used to examine the factors influencing medication literacy. For continuous variables with normal distribution, univariate analysis was conducted using one-way ANOVA. The Kruskal‒Wallis H test was used in ordinal data or nonnormally distributed measures. Ordinal logistic regression was used to analyze the factors affecting the medication literacy scores of MHD patients. Spearman’s correlation analysis was used to analyze the correlation between medication literacy and frailty. A p value of less than 0.05 was considered statistically significant.

## Results

In this study, a total of 297 questionnaires were distributed, and 7 invalid questionnaires were excluded. Ultimately, 290 valid questionnaires were collected, yielding an effective recovery rate of 97.6%.

The demographic characteristics of the participants were shown in Table 1. A total of 290 patients on MHD participated in the study (169 men, 121 women). Most participants were under 65 years old (80.69%).

The medication literacy among 290 MHD respondents was shown in Table 2. The median score was 8.0 (4.0–11.0). Of these, 56 (19.3%) had inadequate medication literacy, 153 (52.8%) had marginal medication literacy, and 81 (27.9%) had adequate medication literacy. The highest accuracy of Item 12 was 239 (82.4%), and the lowest accuracy of Item 14 was 98 (33.8%).

**Table 2:** Results of medication literacy (N = 290)

Items	No. of participants with correct responses (N = 290)	Percentage (%)
**Case scenario 1 : injectable medication for diabetics**		
1 Times per day inject the medicine	148	51.0
2 Doses per morning for syringe	132	45.5
3 Parts of the body for injecting	99	34.1
4 Angle for injecting	163	56.2
5 Identification doctor from prescription	125	43.1
**Case scenario 2 : medication for children**		
6 Doses according to age for Ibuprofen suspension liquid	209	72.1
7 Correct equivalent amount of Ibuprofen suspension liquid	204	70.3
8 Maximum dosage for Ibuprofen suspension liquid	146	50.3
**Case scenario 3 : cold medicine**		
9 Identification of name of medicine	132	45.5
10 Total number of tablets	202	69.7
11 Total of medicine boxes to complete treatment	185	63.8
**Case scenario 4: OTC and Rx drug**		
12 OTC expiry date	239	82.4
13 OTC composition	120	41.4
14 Contraindication	98	33.8

The frailty among 290 MHD respondents was shown in Table 3. The median score was 1.0 (0–2.0). Of these, 86 (29.7%) were nonfrail, 155 (53.4%) were prefrail, and 49 (16.9%) were frail. The highest responses to “yes” for Question 1 were 146 (50.3%), and the lowest responses to “yes” for Question 3 were 50 (17.2%).

**Table 3:** Results of FRAIL Scale (N = 290)

Questions	No. of participants who answered yes	Percentage
1 How much of the time during the past 4 weeks did you feel tired?	146	50.34
2 By yourself and not using aids, do you have any difficulty climbing a flight of stairs without resting?	68	23.45
3 By yourself and not using aids, do you have any difficulty walking a block without stopping?	50	17.24
4 Did a doctor ever tell you that you have more than 5 illness (see list below)? 4 The illnesses include hypertension, diabetes, cancer (other than a minor skin cancer), chronic lung disease, heart attack, congestive heart failure, angina, asthma, arthritis, stroke, and kidney disease.	74	25.52
In the last 1 year, have you unintentionally lost 5% or more of your body weight?	60	20.69

The number of participants with different characteristics at each medication literacy level was listed in Table 4. Sex, age, education, monthly income, caregiver medication assistance, frailty, β2-MG, and s-IP were all associated with medication literacy (P < 0.05). However, primary illness, dialysis vintage, number of medications, comorbidity, BMI, RBC, HGB, FER, ALB, pre-CRE, s-Ca, PTH, and s-K were not associated with medication literacy (P>0.05).

**Table 4:** Results of univariate analysis of medication literacy determinants for patients undergoing hemodialysis (N = 290)

Variable name	Inadequate ML (N = 56)	Marginal ML (N = 153)	Adequate ML (N = 81)	Statistic	*P*
Sex				*χ* 2 = 9.279^c^	0.01
Male	25	87	57		
Female	31	66	24		
Age				Fisher = 38.121^c^	<0.001
<65	29	128	77		
≥ 65	27	25	4		
Education level				H = 54.893^b^	<0.001
Primary school or lower	17	79	42		
Junior high school	2	8	10		
Senior high school	26	58	28		
University or higher	11	8	1		
Monthly income				H = 15.902^b^	<0.001
<3000	30	60	19		
3000 ~ 8000	19	72	38		
>8000	7	21	24		
Primary illness				Fisher = 8.582^c^	0.195
Hypertension	21	65	32		
Diabetes	11	25	8		
Others	22	44	30		
Unknown	2	19	11		
Take medication with the help of a caregiver				*χ* 2 = 31.013^c^	<0.001
Yes	25	19	10		
No	31	134	71		
Dialysis vintage (yrs)				H = 2.633^b^	0.268
<1	4	20	12		
1–5	22	66	36		
>5	30	67	33		
No. of medications				*χ* 2 = 1.745^c^	0.418
< 5	29	91	51		
≥ 5	27	62	30		
Comorbidity				*χ* 2 = 2.853^c^	0.240
< 5	37	112	64		
≥ 5	19	41	17		
Frailty				H = 16.536^b^	<0.001
non-frail	13	44	29		
Pre-frail	20	92	43		
frail	23	17	9		
Body mass index (BMI),m/kg^2^	22.45 ± 4.58	22.67 ± 3.21	22.85 ± 3.12	F = 1.035^d^	0.482
Laboratory findings					
Red blood cell count(RBC), ×10^12^/L	3.78 ± 0.73	3.59 ± 0.67	3.58 ± 0.60	F = 1.085^d^	0.317
Hemoglobin(HGB),g/L	109.05 ± 17.02	106.37 ± 17.23	107.78 ± 15.36	F = 1.203^d^	0.161
Ferritin(FER), ng/mL	158.95 (67.09, 372.40)^a^	262.10 (114.50, 445.55)^a^	216.70 (106.45, 335.30)^a^	H = 230.583^b^	0.477
Albumin(ALB), g/L	41.70 (39.37, 44.08)^a^	43.00 (40.55, 45.65)^a^	44.20 (41.40, 45.70)^a^	H = 33.217^b^	0.156
Pre-dialysis creatinine (pre-CRE), µmol/L	853.43 ± 298.93	945.86 ± 231.35	1049.57 ± 252.12	F = 0.781^d^	0.867
β2-microglobulin (β2-MG) mg/L	50.20 (35.85,61.43)^a^	40.60 (33.96,48.30)^a^	37.90 (30.78, 46.50)^a^	H = 90.519^b^	0.024
Serum calcium (s-Ca), mmol/L	2.22 ± 0.23	2.25 ± 0.22	2.28 ± 0.27	F = 0.997^d^	0.496
Serum inorganic phosphorus(s-IP), mmol/L	1.81 (1.38, 2.26)^a^	1.95(1.65, 2.25)^a^	2.06 (1.83, 2.32)^a^	H = 11.357^b^	0.023
Parathyroid hormone(PTH), pmol/L	42.29 (22.31, 63.49)^a^	46.21 (29.23, 64.32)^a^	48.53 (30.57, 65.74)^a^	H = 107.824^b^	0.541
Serum potassium(s-K),mmol/L	4.81 (4.36, 5.25)^a^	4.69 (4.38, 5.15)^a^	4.71 (4.30, 5.360)^a^	H = 2.610^b^	2.610

^a^Notes: median (IQR)

^b^Kruskal-Wallis H test

^c^chi-square test

^d^One-Way ANOVA.

Ordered logistic regression revealed factors associated with inadequate medication literacy: age (OR = 0.281, 95% CI = 0.139–0.565, p < 0.001 for patients younger than 65); education (OR = 8.612, 95% CI = 3.524–21.046, p < 0.001 for patients with primary school education or lower; OR = 3.405, 95% CI = 1.683–6.887, p = 0.001 for junior high school education); presence of caregiver medication assistance (OR = 2.302, 95% CI = 1.173–4.516, p = 0.015); frailty (OR = 0.440, 95% CI = 0.216–0.893, p = 0.023 for frail patients); and high β2-microglobulin (β2-MG) (OR = 1.010, 95% CI = 1.002–1.019, p = 0.012). (Table 5).

**Table 5:** Results of logistic regression analysis of medication literacy determinants for patients undergoing hemodialysis (N = 290)

Variables	*B*	SE	Wald	*P*	OR	95% Confidence interval for OR	
						Lower	Upper
Sex (refence: Female)							
Male	-0.395	0.258	2.345	0.126	0.674	0.406	1.117
Age (refence:≥65)							
<65	-1.270	0.357	12.649	< 0.001	0.281	0.139	0.565
Monthly income (refence: >8,000)							
< 3,000	0.586	0.400	2.144	0.143	1.797	0.820	3.935
3,000–8,000	0.117	0.352	0.111	0.739	1.124	0.564	2.244
Education level (refence: University or higher)							
Primary school or lower	2.153	0.456	22.308	< 0.001	8.612	3.524	21.046
Junior high school	1.225	0.359	11.619	0.001	3.405	1.683	6.887
Senior high school	0.480	0.367	1.712	0.191	1.617	0.787	3.320
Take medication with the help of a caregiver (refence: No)							
Yes	0.834	0.344	5.881	0.015	2.302	1.173	4.516
Frailty (refence: Frail)							
Not frail	-0.684	0.394	3.023	0.082	0.504	0.233	1.091
Pre-frail	-0.822	0.362	5.168	0.023	0.440	0.216	0.893
β2-microglobulin (β2-MG) mg/L	0.010	0.004	6.285	0.012	1.010	1.002	1.019
Serum inorganic phosphorus(s-IP), mmol/L	-0.388	0.242	2.575	0.109	0.678	0.422	1.090

## Discussion

This study revealed a high prevalence of inadequate and marginal medication literacy among MHD patients, with less than one-third demonstrating adequate literacy. Key factors associated with poorer medication literacy were older age, lower education level, taking medication with the assistance of a caregiver, frail status, and higher β2-MG levels. To our knowledge, this is the first observational study to assess medication literacy, focusing on evaluating Chinese hemodialysis patients’ numeracy and literacy regarding medication labels. The medication literacy status, associated factors and the relationship with frailty were discussed as follows.

### Medication literacy status among MHD patients

Compared to the findings of Xiong et al. [13], this study indicated a higher prevalence of inadequate medication literacy among MHD patients, with only a small portion demonstrating adequate skills. This could be attributed to the use of different medication literacy assessment tools. Xiong et al. [13] employed a 9-item Chinese version of the medication literacy survey questionnaire, which only examined patients’ mastery of medication knowledge and did not assess patients’ active search for medication information and levels of medication skills [6]. The questionnaire could still be improved in assessing medication literacy in diseased or healthy populations [6]. In contrast, the MedLitRxSE questionnaire designed by Sauceda [21] and translated by Zheng et al. used in this study consisted of 14 items [18]. It evaluated patients’ numeracy and literacy regarding medication labels, emphasizing mixed skills.

It was found that MHD patients had the highest accuracy in answering questions about medication expiration dates. This may be because the vast majority of participants in this study were under 65 years old, enabling themselves to read and understand medication labels. In contrast, elderly MHD patients were susceptible to declining cognitive abilities. Comparatively, participants had the lowest accuracy in answering questions about when medications should be stopped, which was consistent with results from Zheng et al. [18]. Patients’ ability to properly understand medication information and make the right choices in case of the occurrence of side effects is essential for medication safety and avoiding harmful health issues. Due to their limited knowledge of medication, MHD patients were at risk of receiving improper and ineffective treatment. The numerous side effects and interactions between the multiple medications used by these patients further increased the risk of medication-related issues [22]. Our study thus emphasized the importance of improving medication literacy among MHD patients.

### The associated factors of medication literacy in MHD patients

Many studies have confirmed that age is an influencing factor for medication literacy [7, 8, 23, 24]. Due to the decreasing cognitive and literacy capacities in elderly patients, health self-management involving understanding new information may become increasingly challenging [25]. Furthermore, compared to younger individuals, elderly individuals have poorer learning and recollection abilities owing to deteriorating memory and physical ability. This leads to an inadequate grasp of medication-related knowledge and skills, ultimately resulting in lower medication literacy.

This study demonstrated that patients without formal education and with primary or junior education had lower medication literacy than those with higher education. Many studies have confirmed the influence of education levels on patients’medication literacy [8, 26, 27]. Individuals with higher education levels tend to have greater health awareness and are more proactive in learning medication use. Additionally, they have better access to medication information from sources such as the Internet and written materials.

Our regression analysis indicated that MHD patients who had caregiver assistance had lower medication literacy. This may be because patients overly rely on caregivers, leading to insufficient motivation to actively learn medication knowledge themselves. Although social support plays a key role in helping chronically ill patients self-manage their medication use [11] and MHD patients with positive support systems from healthcare providers were more successful in adhering to their medication regimen [28, 29], over-support from caregivers can lead to dependence which is not conducive to self-management. Therefore, while providing support, caregivers need to moderately guide patients to learn medication knowledge themselves, ensuring that patients take initiative to improve their own medication literacy. Additionally, family support should also avoid over-supporting patients, and appropriately encourage self-management of medications. In summary, while supporting patients, healthcare providers and families need to guide patients to learn independently as well, thereby improving patients’medication knowledge and self-management capabilities.

Using Spearman’s correlation analysis, the present study found that the state of frailty was negatively correlated with the level of medication literacy, which was consistent with existing research in patients with coronary heart disease [15]. This may be because inadequate medication literacy could lead to suboptimal treatment compliance and efficacy, improper medication use, negative drug and appetite interactions [30], poor disease control, and ultimately worsened nutritional status [15, 30]. Moreover, Cao et al. revealed that health literacy was associated with frailty in MHD patients [31]. Building upon this, medication literacy, as an application of health literacy in pharmacy practice, may also be related to frailty.

Logistic regression analysis also showed that prefrailty was an independent predictor of medication literacy in our study. Frailty indicated a decline in function, cognition and health. Patients with prefrailty were at risk of developing frailty, but effective interventions could prevent it. To maintain their health and relieve discomfort, prefrail patients may utilize self-medication and adhere to treatment regimens prescribed by their doctors, such as accurately following medication instructions. If they develop additional diseases, their medical prescriptions will increase, which requires greater medication literacy. This could explain why prefrailty patients have protective factors for medication literacy levels.

Hemodialysis is a common method for purifying blood. It could remove small molecule toxins in patients with uremia effectively, but the removal of middle molecule toxins such as β2-MG was less effective [32]. High levels of these toxins in their body could suppress appetite and even cause nausea and vomiting, leading to insufficient intake and an increased risk of malnutrition [33]. Previous studies have shown that patients at risk of malnutrition had lower medication literacy [14]. As a result, there was a link between patients’ β2-MG levels and their medication literacy.

The strength of our study lies in the fact that it showed a correlation between prefrailty, β2-MG and medication literacy, despite most previous studies indicating that medication literacy was a risk factor for frailty. Our study deepened the understanding of how a patient’s physical health could impact their medication literacy.

### Limitations and future research

Our study has several limitations. First, approximately 80% of our participants were under 65 years old. Second, this was a single-center study with a small sample size, limiting the generalizability of our results. To address these limitations, further studies should be multicenter and have larger sample sizes. Interventional studies should also be conducted based on our results to improve patients’ medication literacy levels. In addition, future research should include follow-up evaluations of medication literacy and track the factors that influence it over time.

## Conclusions

There is still a need for improved medication literacy among MHD patients. Age, education level, caregiver assistance, β2-MG levels, and prefrailty are associated with medication literacy. The correlation between frailty and poorer medication literacy implies that frail patients are an especially high-risk group in need of medication literacy support. The link with higher β2-MG suggests medication literacy may decline with worsening kidney function. This can guide clinical practice to provide targeted interventions and education to improve medication literacy and adherence in MHD patients. Future research can explore optimal educational strategies and training programs to boost medication literacy in this vulnerable population.

## Data Availability

Data available on request from the corresponding author.

## Abbreviations

ALB: Albumin
ESRD: End-stage renal disease
FER: Ferritin
HGB: Hemoglobin
MHD: Maintenance hemodialysis
pre-CRE: Predialysis creatinine
PTH: Parathyroid hormone
RBC: Red blood cell count
s-Ca: Serum calcium
s-IP: Phosphorus
s-K: Serum potassium
β2-MG: β2-microglobulin
